# Mutations of mitochondrial genome in carotid atherosclerosis

**DOI:** 10.3389/fgene.2015.00111

**Published:** 2015-03-19

**Authors:** Margarita A. Sazonova, Andrey V. Zhelankin, Valeria A. Barinova, Vasily V. Sinyov, Zukhra B. Khasanova, Anton Y. Postnov, Alexander N. Orekhov, Yuri V. Bobryshev, Igor A. Sobenin

**Affiliations:** ^1^Laboratory of Angiopathology, Institute of General Pathology and PathophysiologyMoscow, Russia; ^2^Laboratory of Medical Genetics, Russian Cardiology Research and Production ComplexMoscow, Russia; ^3^Institute for Atherosclerosis Research, Skolkovo Innovative CentreMoscow, Russia; ^4^Department of Biophysics, Biological Faculty, Moscow State UniversityMoscow, Russia; ^5^Faculty of Medicine, School of Medical Sciences, University of New South WalesSydney, NSW, Australia

**Keywords:** atherosclerosis, mitochondrial genome, mutation, next generation sequencing, mitochondria

## Abstract

With aim of detection the spectrum of mitochondrial DNA mutations in patients with carotid atherosclerosis from Moscow Region, we used a Roche 454 high-throughput sequencing of the whole mitochondrial genome. We have found that the presence of a number of homoplasmic mitochondrial DNA mutations in genes of 16S ribosomal RNA, subunits 2, 4, and 5 NADH dehydrogenase, subunits 1 and 2 cytochrome C oxidase, subunit 6 ATP-synthase, tRNA- Leu 2 and cytochrome B differed between conventionally healthy participants of the study and patients with carotid atherosclerosis. We also found heteroplasmic mutations, including insertions one or several nucleotides, that occurred more frequently in mitochondrial DNA of conventionally healthy participants of the study or patients with atherosclerotic lesions.

## Introduction

Mutations of mitochondrial genome are known nowadays as one of the genetic risk factors of different multifactorial human pathologies, including cardiovascular diseases such as CHD, myocardial infarction, and stroke (Kofler et al., 2009; Jia et al., 2013; Szabó, 2013). Since the major morphological basis of these diseases is atherosclerotic vascular lesions, it is possible to assume the association of changes in the human mitochondrial genome with the risk of atherosclerosis. Recent studies made by our scientific group showed that a number of mtDNA mutations are associated with the presence of atherosclerotic plaques and subclinical carotid atherosclerosis (Wallace, 1994; Kofler et al., 2009). The penetrance and expression of mitochondrial mutations may vary greatly between relatives and depends mainly on a genotype and the level of heteroplasmy (a ratio of mutant and normal copies of mitochondrial genome). Therefore, a quantitative assessment of a mutant allele of mitochondrial genome is necessary for studying the association of mitochondrial mutations with atherosclerosis.

Mitochondrial mutations may result in defects in the protein chains of respiratory enzymes and tRNAs that are synthesized in mitochondria, which probably lead to changes in mitochondrial function. However, the mechanisms of association of mitochondrial mutations with the formation of atherosclerotic lesions in the vascular wall still remain obscure. The present study based on high-througput sequencing of the whole human mitochondrial genome was carried out to test the hypothesis that mtDNA mutations can be associated with an increased risk of atherosclerosis.

## Materials and methods

Material for this study was collected in accordance with the principles outlined in the Declaration of Helsinki and informed, written consent was obtained from each patient/donor. The study was approved by the Institutional Review Board of Institute of General Pathology and Pathophysiology, Moscow, Russian Federation; Russian Cardiology Research and Production Complex, Moscow and Institute for Atherosclerosis Research, Skolkovo Innovative Centre, Moscow, Russian Federation.

The studied sample included 60 individuals from Moscow region [25 male (m) and 35 female (f)] with an average age of 64.0 years (m: 63.5, f: 64.3) (Supplemental Tables 1–3). 30 patients (m: 14, f: 16) with an average age of 65.6 years were characterized by a presence of ultrasonographically detected atherosclerotic lesions in carotid arteries. The number of conventionally healthy participants of the study was 30 (m: 11, f: 19) with an average age of 62.4 years.

To evaluate the state of the carotid artery wall, high-resolution B-mode ultrasonography was performed with ultrasound scanner SonoScape SSI-1000 (China). Borderline carotid intima-media thickness (CIMT) values for Moscow region were used to characterize the presence of carotid atherosclerosis (Sobenin et al., 2011). Persons with the presence of atherosclerotic plaque or thickening of the intima-media layer exceeding the boundaries of the 75th percentile, as well as the combination of these factors, were considered as patients with atherosclerosis (mean CIMT 1.04 ± 0.02, m: 1.01 ± 0.02, f: 1.07 ± 0.03 mm). Conventionally healthy participants of the study were characterized by CIMT values which do not exceed median values for appropriate age group and by the absence of atherosclerotic plaques (mean CIMT 0.69 ± 0.02, m: 0.75 ± 0.02, f: 0.66 ± 0.03 mm).

Total DNA was extracted from whole blood by a phenol-chloroform method with proteinase K lysis. An enrichment of mtDNA was performed using Qiagen™ REPLI-g Mitochondrial Kit. The amount of DNA was about 50 ng per 50 μl of reaction buffer and the yield of enriched fraction was 5 μg per reaction. The resulting enriched fraction contained DNA fragments of size 10–15 kB.

Roche 454 GS Junior Titanium system (Roche Diagnostics GmbH) was used to carry out a high-throughput sequencing of mitochondrial genome. 500 ng of enriched mtDNA fraction was taken for creation a shotgun DNA fragments library and for further sequencing. Sample preparation for sequencing was carried out in accordance with the manufacturer's recommendations. Instrumental run and analysis of the sequencing quality were performed using GS Sequencer and GS Run Browser software (Roche Diagnostics GmbH).

Analysis of mitochondrial DNA and SNP detection was performed using GS Reference Mapper software (Roche Diagnostics GmbH). The revised Cambridge reference (rCRS) of human mitochondrial genome NC_012920.1 was used for mapping (Andrews et al., 1999). A statistical data analysis was performed using Microsoft Excel 2010 and IBM SPSS Statistics v.21.0 software (http://spss.ru.joydownload.com/&c=20?gclid=COTnxtbesLwCFaHbcgodiiMAtQ).

## Results and discussion

**Figure 1:**
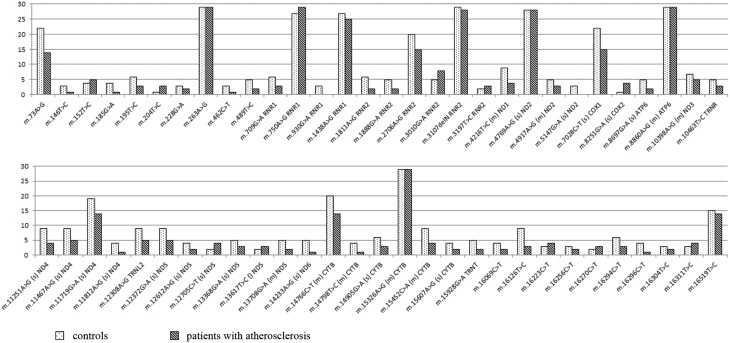
**Frequency of the most common homoplasmic mtDNA mutations**. For single nucleotide substitutions in coding region there is a designation of the character of mutation (s, synonymous; m, missence), and corresponding gene.

A pilot whole genome sequencing using 454 Roche GS Junior Titanium system gave about 70-fold average coverage of mtDNA which allowed to detect in the observed sample a total of 422 homoplasmic mitochondrial genome variants that differed from rCRS. In the investigated samples we distinguished 59 mitochondrial genome mutations (Table 1). They were present in over 10% of the total sample. Some of these mutations occurred in over 10% of the sample of patients with atherosclerosis. Meanwhile some mutations were more characteristic for over 10% of conventionally healthy participants of the study (a control group) (Figure 1). All these mutations are known and were reported before as markers of appropriate mitochondrial haplogroups (van Oven and Kayser, 2009). Most of these mutations (about 70%) were characterized by the higher prevalence in the control group than in patients with atherosclerosis. As we used rCRS as a reference, all mutations that were found in this study can be named according to HGVS nomenclature using prefix NC_012920.1. Mitochondrial mutations m.930G>A and m.5147G>A were found only in the control group. The presence of mutations m.14233A>G, m.185G>A, m.11812A>G, m.14798T>C, m.16296C>T, m.146T>C, m.462C>T, m.1811A>G, m.16126T>C, m.489T>C, m.1888G>A, m.8697G>A, m.13708G>A, m.15928G>A, m.4216T>C, m.11251A>G, m.15452C>A, m.195T>C, m.709G>A, m.12612A>G, m.14905G>A, m.15607A>G, m.16069C>T, and m.16294C>T was from 2 to 5-fold higher in control group than in patients with atherosclerosis. This fact allows us to assume anti-atherogenic character of these mutations. Single nucleotide replacements m.8251G>A, m.204T>C, m.12705C>T, and m.3010G>A were observed 1.6-fold more often in atherosclerotic patients compared to the control group.

**Table 1:** **Common homoplasmic mutations, identified in the study**.

**Position**	**Gene/Region**	**Character (m, missense; s, synonymous), for mutations in protein genes**	**Name**	**Sum in general**	**Sum in controls**	**Sum in atherosclerotic patients**
73	Noncoding	–	m.73A>G	36	22	14
146	Noncoding	–	m.146T>C	4	3	1
152	Noncoding	–	m.152T>C	9	4	5
185	Noncoding	–	m.185G>A	5	4	1
195	Noncoding	–	m.195T>C	9	6	3
204	Noncoding	–	m.204T>C	4	1	3
228	Noncoding	–	m.228G>A	5	3	2
263	Noncoding	–	m.263A>G	58	29	29
462	Noncoding	–	m.462C>T	4	3	1
489	Noncoding	–	m.489T>C	7	5	2
709	RNR1	–	m.709G>A	9	6	3
750	RNR1	–	m.750A>G	56	27	29
930	RNR1	–	m.930G>A	3	3	0
1438	RNR1	–	m.1438A>G	52	27	25
1811	RNR2	–	m.1811A>G	8	6	2
1888	RNR2	–	m.1888G>A	7	5	2
2706	RNR2	–	m.2706A>G	35	20	15
3010	RNR2	–	m.3010G>A	13	5	8
3107	RNR2	–	m.3107delN	57	29	28
3197	RNR2	–	m.3197T>C	5	2	3
4216	ND1	m	m.4216T>C	13	9	4
4769	ND2	s	m.4769A>G	56	28	28
4917	ND2	m	m.4917A>G	8	5	3
5147	ND2	s	m.5147G>A	3	3	0
7028	COX1	s	m.7028C>T	37	22	15
8251	COX2	s	m.8251G>A	5	1	4
8697	ATP6	s	m.8697G>A	7	5	2
8860	ATP6	m	m.8860A>G	58	29	29
10398	ND3	m	m.10398A>G	12	7	5
10463	TRNR	–	m.10463T>C	8	5	3
11251	ND4	s	m.11251A>G	13	9	4
11467	ND4	s	m.11467A>G	14	9	5
11719	ND4	s	m.11719G>A	33	19	14
11812	ND4	s	m.11812A>G	5	4	1
12308	TRNL2	–	m.12308A>G	14	9	5
12372	ND5	s	m.12372G>A	14	9	5
12612	ND5	s	m.12612A>G	6	4	2
12705	ND5	s	m.12705C>T	6	2	4
13368	ND5	s	m.13368G>A	8	5	3
13617	ND5	s	m.13617T>C	5	2	3
13708	ND5	m	m.13708G>A	7	5	2
14233	ND6	s	m.14233A>G	6	5	1
14766	CYTB	m	m.14766C>T	34	20	14
14798	CYTB	m	m.14798T>C	5	4	1
14905	CYTB	s	m.14905G>A	9	6	3
15326	CYTB	m	m.15326A>G	58	29	29
15452	CYTB	m	m.15452C>A	13	9	4
15607	CYTB	s	m.15607A>G	6	4	2
15928	TRNT	–	m.15928G>A	7	5	2
16069	Noncoding	–	m.16069C>T	6	4	2
16126	Noncoding	–	m.16126T>C	12	9	3
16223	Noncoding	–	m.16223C>T	7	3	4
16256	Noncoding	–	m.16256C>T	5	3	2
16270	Noncoding	–	m.16270C>T	5	2	3
16294	Noncoding	–	m.16294C>T	9	6	3
16296	Noncoding	–	m.16296C>T	5	4	1
16304	Noncoding	–	m.16304T>C	5	3	2
16311	Noncoding	–	m.16311T>C	7	3	4
16519	Noncoding	–	m.16519T>C	29	15	14

We also found 104 heteroplasmic mutations. Seventy two mutations were located in the coding regions of mtDNA. It is necessary to say that among them there were 15 insertions and no deletions. 19 of heteroplasmic mutations were found also in homoplasmic state. 53 mutations (22 synonymous, 22 missense, 1 nonsense, and 8 insertions) were located in genes of respiratory chain enzymes. Most of heteroplasmic mutations were rare variants found only in one individual. However, we found 14 most common heteroplasmic mitochondrial genome mutations in this sample (Table 2). Mutations m.73A>G, m.295C>T, m.4216T>C, m.9477G>A, m.15924A>G, m.16311T>C, m.576insC, m.8516insA, m.8516insC, m.8528insA, m.8930insG, m.10958insC, m.13047insC, and m.13050insC were found both in the control group and in patients with atherosclerosis. There were no statistically significant differences in the occurrence and mean heteroplasmy level of these mutations between the compared groups, but mutations m.8516insA, m.8516insC, m.8930insG, m.10958insC, and m.13050insC occurred more frequently in the control group than in atherosclerotic patients with heteroplasmy level from 6 to 41%. Missense mutation m.9477G>A in MT-COX3 gene and insertion m.8528insA in MT-ATP8 occurred more frequently in patients with atherosclerotic lesions.

**Table 2:**
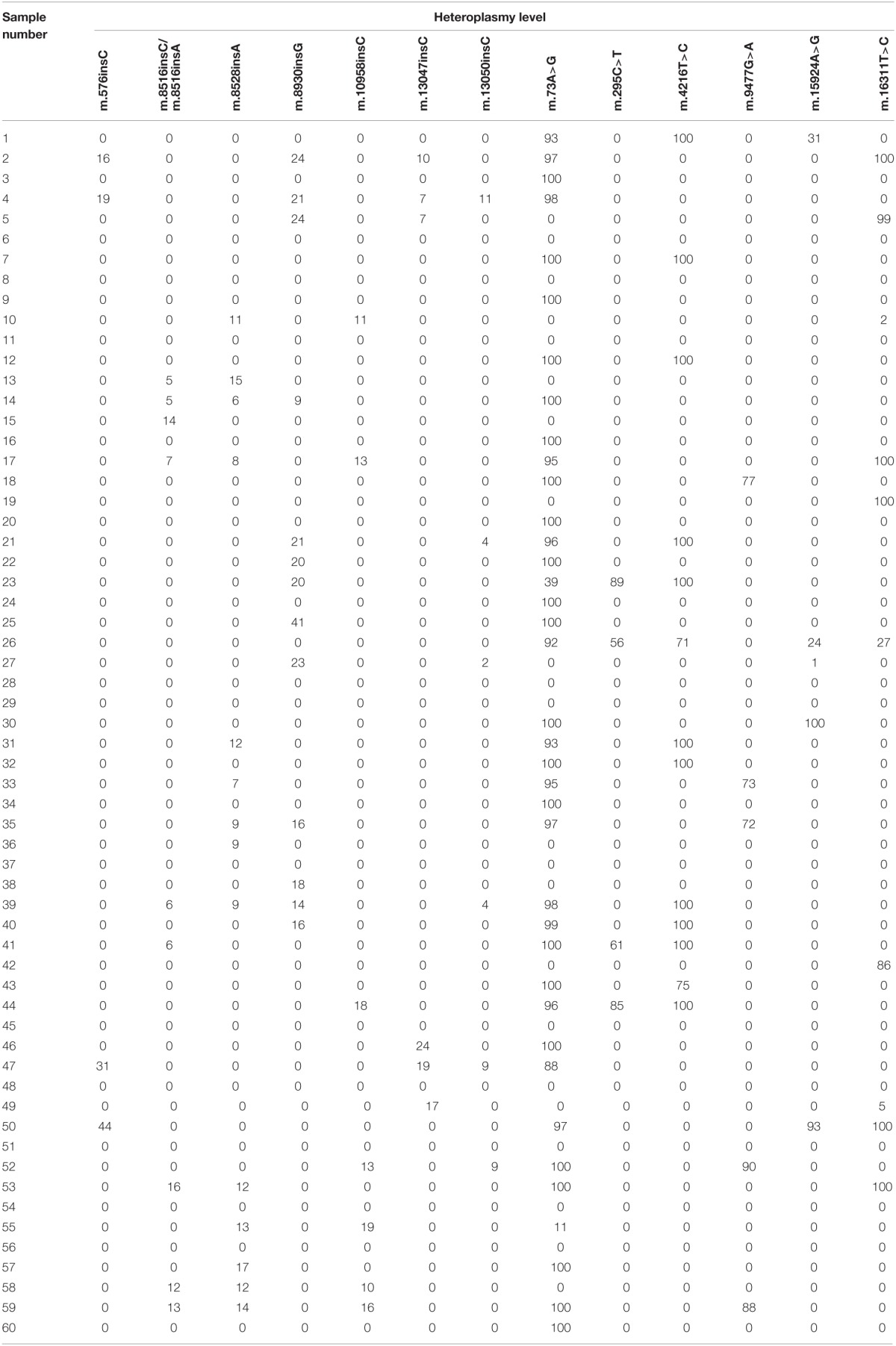
**Common heteroplasmic mutations, identified in the study**.

Previously, using pyrosequencing of mtDNA fragments with PSQ 96MA system (Biotage, Sweden) we have found 11 heteroplasmic mutations associated with atherosclerosis: m.652delG, m.1555A>G, m.3256C>T, m.3336T>C, m.652insG, m.5178C>A, m.12315G>A, m.14459G>A, m.13513G>A, m.14846G>A and m.15059G>A, using NC_012920.1 as a reference (Sazonova et al., 2009, 2014; Sobenin et al., 2013). In the present study these mutations were not detected. It can be possibly related with differences in sample preparation of the compared methods. Particularly, 454 sequencing requires high amounts of DNA per one experiment, and to obtain the sufficient quantity of mtDNA there is a necessity to carry out whole mitochondrial genome amplification or long-range PCR. This stage can produce specific amplification of random mtDNA molecules and complicate further detection of a number of mutations with low heteroplasmy level.

In the present study with the help of next generation sequencing technology, specifically, using 454 Roche GS Junior Titanium system, 422 homoplasmic and 104 heteroplasmic mitochondrial genome mutations were detected. We have found that the presence of a number of homoplasmic mitochondrial DNA mutations in genes of 16S ribosomal RNA, subunits 2, 4, and 5 NADH dehydrogenase, subunits 1 and 2 cytochrome C oxidase, subunit 6 ATP-synthase, tRNA- Leu 2 and cytochrome B differed between conventionally healthy participants of the study and patients with carotid atherosclerosis. We also found heteroplasmic mutations, including insertions one or several nucleotides, that occurred more frequently in mitochondrial DNA of conventionally healthy participants of the study or patients with atherosclerotic lesions.

It is necessary to mention that homoplasmic single nucleotide replacements m.8251G>A, m.204T>C, m.12705C>T, m.3010G>A, heteroplasmic missense mutation m.9477G>A and insertion m.8528insA occurred more frequently in patients with atherosclerotic lesions compared to the control group. The findings of this study can be useful for medical geneticists and scientists, carrying out researches in the field of cardiovascular pathologies and atherogenesis.

### Conflict of interest statement

The authors declare that the research was conducted in the absence of any commercial or financial relationships that could be construed as a potential conflict of interest.
