# Supplementary material for: Mutations of mitochondrial genome in carotid atherosclerosis
Source: Front Genet. 2015 Mar 19;6:111. doi: 10.3389/fgene.2015.00111 (PMC4365735; doi:10.3389/fgene.2015.00111)
Supplement: Supplementary file 1 [file Table1.DOCX]

**Supplemental TABLE 1.** Relevant data about samples/subjects analysed in the study

| Sample number | Atherosclerosis presence | Gender  (1- male,  2 - female) | Age, years | Height, cm | Weight, kg | BMI, kg/m^2^ | Systolic blood pressure,mm Hg | Diastolic blood pressure, mm Hg | Pulse, bpm |
| --- | --- | --- | --- | --- | --- | --- | --- | --- | --- |
| 1 | 0 | 1 | 65 | 175 | 85,2 | 27,8 | 145 | 90 | 69 |
| 2 | 0 | 1 | 64 | 176 | 92,8 | 30 | 156 | 92 | 100 |
| 3 | 0 | 1 | 57 | 169 | 84,2 | 29,5 | 140 | 88 | 89 |
| 4 | 0 | 1 | 61 | 174 | 81 | 26,8 | 140 | 90 | 76 |
| 5 | 1 | 1 | 66 | 168 | 61 | 21,6 | 158 | 86 | 69 |
| 6 | 1 | 1 | 70 | 174 | 60 | 19,8 | 139 | 70 | 73 |
| 7 | 1 | 1 | 64 | 176 | 73 | 23,6 | 149 | 86 | 78 |
| 8 | 1 | 1 | 61 | 172 | 98,8 | 33,4 | 140 | 97 | 90 |
| 9 | 0 | 2 | 63 | 164 | 74 | 27,5 | 124 | 70 | 67 |
| 10 | 0 | 2 | 73 | 157 | 61 | 24,7 | 103 | 78 | 77 |
| 11 | 0 | 2 | 56 | 164 | 63,4 | 23,6 | 118 | 73 | 73 |
| 12 | 0 | 2 | 58 | 162 | 56,4 | 21,5 | 124 | 86 | 88 |
| 13 | 0 | 2 | 69 | 160 | 67,2 | 26,3 | 113 | 79 | 83 |
| 14 | 0 | 2 | 64 | 167 | 62 | 22,2 | 127 | 93 | 89 |
| 15 | 1 | 2 | 71 | 164 | 78 | 29 | 131 | 87 | 73 |
| 16 | 1 | 2 | 64 | 168 | 68 | 24,1 | 146 | 92 | 83 |
| 17 | 1 | 2 | 64 | 164 | 78,5 | 29,2 | 128 | 74 | 81 |
| 18 | 1 | 2 | 70 | 161 | 65,5 | 25,3 | 123 | 71 | 75 |
| 19 | 1 | 1 | 63 | 169 | 74 | 25,9 | 124 | 70 | 73 |
| 20 | 0 | 1 | 42 | 182 | 90 | 27,2 | 125 | 80 | 69 |
| 21 | 0 | 2 | 55 | 155 | 73,4 | 30,6 | 147 | 94 | 78 |
| 22 | 0 | 2 | 76 | 164 | 77 | 28,6 | 155 | 85 | 90 |
| 23 | 0 | 2 | 54 | 155 | 60,2 | 25,1 | 126 | 91 | 80 |
| 24 | 0 | 2 | 70 | 170 | 70,2 | 24,3 | 133 | 92 | 76 |
| 25 | 0 | 2 | 60 | 157 | 68 | 27,6 | 120 | 80 | 73 |
| 26 | 1 | 2 | 69 | 160 | 71 | 27,7 | 130 | 65 | 76 |
| 27 | 1 | 2 | 65 | 156 | 82 | 33,7 | 122 | 72 | 66 |
| 28 | 1 | 2 | 54 | 168 | 62 | 22 | 120 | 68 | 63 |
| 29 | 1 | 2 | 52 | 165 | 89,2 | 32,8 | 123 | 78 | 77 |
| 30 | 1 | 2 | 60 | 154 | 64,8 | 27,3 | 149 | 96 | 86 |
| 31 | 1 | 1 | 71 | 170 | 83,5 | 28,9 | 139 | 76 | 55 |
| 32 | 1 | 1 | 51 | 187 | 117,4 | 33,6 | 161 | 93 | 82 |
| 33 | 1 | 1 | 69 | 180 | 91,2 | 28,1 | 135 | 82 | 52 |
| 34 | 1 | 1 | 68 | 170 | 71 | 24,6 | 139 | 85 | 60 |
| 35 | 1 | 1 | 63 | 179 | 138 | 43,1 | 153 | 89 | 97 |
| 36 | 1 | 1 | 64 | 174 | 72,6 | 24 | 137 | 85 | 61 |
| 37 | 1 | 1 | 61 | 164 | 66 | 24,5 | 155 | 101 | 76 |
| 38 | 0 | 1 | 67 | 178 | 74 | 23,4 | 145 | 71 | 53 |
| 39 | 0 | 1 | 59 | 176 | 90,8 | 29,3 | 137 | 88 | 68 |
| 40 | 0 | 1 | 71 | 166 | 72 | 26,1 | 153 | 73 | 68 |
| 41 | 0 | 1 | 69 | 176 | 99,6 | 32,2 | 155 | 82 | 73 |
| 42 | 0 | 2 | 59 | 152 | 53,4 | 23,1 | 130 | 81 | 82 |
| 43 | 0 | 2 | 64 | 176 | 63,4 | 20,5 | 128 | 93 | 74 |
| 44 | 0 | 2 | 69 | 150 | 62,8 | 27,9 | 150 | 70 | 76 |
| 45 | 0 | 2 | 60 | 163 | 70,8 | 26,6 | 120 | 81 | 63 |
| 46 | 0 | 2 | 57 | 166 | 56,6 | 20,5 | 132 | 77 | 69 |
| 47 | 1 | 2 | 67 | 153 | 63,3 | 27 | 128 | 84 | 65 |
| 48 | 1 | 2 | 70 | 153 | 71 | 30,3 | 132 | 66 | 77 |
| 49 | 1 | 2 | 74 | 161 | 82,3 | 31,8 | 142 | 82 | 59 |
| 50 | 1 | 2 | 63 | 158 | 69 | 27,6 | 140 | 90 | 73 |
| 51 | 0 | 1 | 52 | 176 | 74 | 23,9 | 129 | 82 | 68 |
| 52 | 0 | 1 | 71 | 173 | 83,2 | 27,8 | 144 | 91 | 67 |
| 53 | 0 | 2 | 71 | 163 | 66,2 | 24,9 | 120 | 75 | 72 |
| 54 | 1 | 1 | 69 | 166 | 70,2 | 25,5 | 153 | 83 | 75 |
| 55 | 1 | 1 | 70 | 174 | 82 | 27,1 | 153 | 90 | 64 |
| 56 | 1 | 2 | 75 | 155 | 63 | 26,2 | 149 | 54 | 92 |
| 57 | 1 | 2 | 70 | 170 | 80 | 27,7 | 134 | 74 | 76 |
| 58 | 1 | 2 | 70 | 160 | 66,2 | 25,9 | 169 | 90 | 76 |
| 59 | 0 | 2 | 50 | 151 | 56,2 | 24,6 | 122 | 86 | 68 |
| 60 | 0 | 2 | 65 | 157 | 73 | 29,6 | 163 | 95 | 81 |
