# Supplementary material for: Mutations of mitochondrial genome in carotid atherosclerosis
Source: Front Genet. 2015 Mar 19;6:111. doi: 10.3389/fgene.2015.00111 (PMC4365735; doi:10.3389/fgene.2015.00111)
Supplement: Supplementary file 2 [file Table2.DOCX]

**Supplemental TABLE 2.** Relevant data about samples/subjects analysed in the study

| Sample number | Smoking | Smoking status, years | Alcohol consumption | Diabetes mellitus | Hypertension | Myocardial infarction | Stroke |
| --- | --- | --- | --- | --- | --- | --- | --- |
| 1 | 0 | 0 | 0 | 0 | 0 | 0 | 0 |
| 2 | 1 | 15 | 0 | 0 | 0 | 0 | 0 |
| 3 | 0 | 0 | 0 | 0 | 0 | 0 | 0 |
| 4 | 0 | 0 | 0 | 0 | 0 | 0 | 0 |
| 5 | 0 | 14 | 0 | 0 | 0 | 0 | 0 |
| 6 | 0 | 0 | 0 | 0 | 0 | 0 | 0 |
| 7 | 1 | 48 | 1 | 0 | 0 | 0 | 0 |
| 8 | 0 | 6 | 0 | 0 | 0 | 0 | 0 |
| 9 | 0 | 0 | 0 | 0 | 0 | 0 | 0 |
| 10 | 0 | 0 | 0 | 0 | 0 | 0 | 0 |
| 11 | 0 | 0 | 0 | 0 | 0 | 0 | 0 |
| 12 | 0 | 0 | 0 | 0 | 0 | 0 | 0 |
| 13 | 0 | 0 | 0 | 0 | 0 | 0 | 0 |
| 14 | 0 | 0 | 0 | 0 | 0 | 0 | 0 |
| 15 | 0 | 0 | 0 | 0 | 0 | 0 | 0 |
| 16 | 0 | 0 | 0 | 0 | 0 | 0 | 0 |
| 17 | 0 | 0 | 0 | 0 | 0 | 0 | 0 |
| 18 | 0 | 0 | 0 | 0 | 0 | 0 | 0 |
| 19 | 0 | 0 | 0 | 0 | 0 | 0 | 0 |
| 20 | 0 | 0 | 0 | 0 | 1 | 0 | 0 |
| 21 | 1 | 35 | 0 | 0 | 0 | 0 | 0 |
| 22 | 0 | 0 | 0 | 0 | 0 | 0 | 0 |
| 23 | 0 | 0 | 0 | 0 | 0 | 0 | 0 |
| 24 | 0 | 0 | 0 | 0 | 0 | 0 | 0 |
| 25 | 0 | 0 | 0 | 1 | 0 | 0 | 0 |
| 26 | 0 | 40 | 0 | 0 | 0 | 0 | 0 |
| 27 | 0 | 0 | 0 | 0 | 0 | 0 | 0 |
| 28 | 0 | 0 | 0 | 0 | 0 | 0 | 0 |
| 29 | 0 | 0 | 0 | 0 | 0 | 0 | 0 |
| 30 | 0 | 0 | 0 | 1 | 0 | 0 | 0 |
| 31 | 0 | 12 | 0 | 0 | 0 | 0 | 1 |
| 32 | 1 | 33 | 0 | 0 | 1 | 0 | 0 |
| 33 | 0 | 0 | 0 | 0 | 1 | 0 | 0 |
| 34 | 0 | 37 | 0 | 0 | 1 | 0 | 0 |
| 35 | 1 | 45 | 0 | 0 | 1 | 0 | 1 |
| 36 | 1 | 50 | 1 | 0 | 1 | 0 | 0 |
| 37 | 0 | 0 | 0 | 0 | 0 | 0 | 0 |
| 38 | 0 | 0 | 0 | 0 | 0 | 0 | 0 |
| 39 | 0 | 0 | 0 | 0 | 1 | 0 | 0 |
| 40 | 0 | 0 | 0 | 0 | 1 | 0 | 0 |
| 41 | 0 | 40 | 0 | 0 | 1 | 0 | 0 |
| 42 | 0 | 0 | 0 | 0 | 0 | 0 | 0 |
| 43 | 0 | 0 | 0 | 0 | 0 | 0 | 0 |
| 44 | 0 | 15 | 0 | 0 | 1 | 0 | 0 |
| 45 | 0 | 0 | 0 | 0 | 1 | 0 | 0 |
| 46 | 0 | 0 | 0 | 0 | 0 | 0 | 0 |
| 47 | 0 | 0 | 0 | 0 | 1 | 0 | 0 |
| 48 | 0 | 0 | 0 | 0 | 1 | 0 | 0 |
| 49 | 0 | 0 | 0 | 0 | 1 | 0 | 0 |
| 50 | 0 | 0 | 0 | 0 | 1 | 0 | 0 |
| 51 | 0 | 0 | 0 | 0 | 0 | 0 | 0 |
| 52 | 1 | 21 | 1 | 0 | 1 | 0 | 0 |
| 53 | 0 | 0 | 0 | 0 | 1 | 0 | 0 |
| 54 | 0 | 0 | 0 | 0 | 1 | 0 | 1 |
| 55 | 0 | 0 | 0 | 0 | 1 | 0 | 0 |
| 56 | 0 | 0 | 0 | 0 | 0 | 0 | 0 |
| 57 | 0 | 0 | 0 | 0 | 0 | 0 | 0 |
| 58 | 0 | 11 | 0 | 0 | 1 | 1 | 1 |
| 59 | 0 | 3 | 0 | 0 | 0 | 0 | 0 |
| 60 | 0 | 0 | 0 | 0 | 1 | 0 | 0 |
