# Supplementary material for: Mutations of mitochondrial genome in carotid atherosclerosis
Source: Front Genet. 2015 Mar 19;6:111. doi: 10.3389/fgene.2015.00111 (PMC4365735; doi:10.3389/fgene.2015.00111)
Supplement: Supplementary file 3 [file Table3.DOCX]

**Supplemental TABLE 3.** Relevant data about samples/subjects analysed in the study

| Sample number | Cholesterol, mM | Triglycerides, mM | HDL Cholesterol, mM | LDL Cholesterol, mM | Atherogenic index | Plaque  (0 - no plaque,  1 - plaque with stenosis under 20%, 2 - stenosis 20-50%,  3 - stenosis over 50%) | Statins | IMT mean, mm | Plaque presence |
| --- | --- | --- | --- | --- | --- | --- | --- | --- | --- |
| 1 | 5,04 | 0,76 | 0,87 | 3,81 | 4,4 | 0 | 0 | 0,79 | 0 |
| 2 | 8,18 | 0,99 | 1,43 | 6,28 | 4,4 | 0 | 0 | 0,69 | 0 |
| 3 | 5,4 | 0,82 | 1,71 | 3,3 | 1,9 | 1 | 1 | 0,71 | 1 |
| 4 | 4,81 | 1,34 | 1,32 | 2,85 | 2,1 | 0 | 0 | 0,8 | 0 |
| 5 | 4,7 | 0,99 | 2,16 | 2,07 | 1 | 2 | 0 | 1,02 | 1 |
| 6 | 7,35 | 1,62 | 1,93 | 4,66 | 2,4 | 2 | 0 | 0,99 | 1 |
| 7 | 3,51 | 1,18 | 1,38 | 1,56 | 1,1 | 3 | 0 | 1,03 | 1 |
| 8 | 7,71 | 2,45 | 1,48 | 5,08 | 3,4 | 2 | 0 | 0,91 | 1 |
| 9 | 5,66 | 1,21 | 1,63 | 3,46 | 2,1 | 0 | 0 | 0,74 | 0 |
| 10 | 6,42 | 0,77 | 1,2 | 4,85 | 4 | 0 | 0 | 0,77 | 0 |
| 11 | 6,73 | 0,96 | 2,56 | 3,71 | 1,4 | 0 | 0 | 0,61 | 0 |
| 12 | 7,14 | 1,01 | 1,87 | 4,8 | 2,6 | 0 | 0 | 0,57 | 0 |
| 13 | 7,06 | 1,65 | 1,5 | 4,78 | 3,2 | 1 | 0 | 0,55 | 1 |
| 14 | 7,77 | 1,24 | 1,39 | 5,79 | 4,2 | 0 | 0 | 0,66 | 0 |
| 15 | 5,82 | 0,86 | 1,87 | 3,54 | 1,9 | 2 | 0 | 1,14 | 1 |
| 16 | 7,06 | 1,04 | 1,23 | 5,34 | 4,3 | 1 | 0 | 0,98 | 1 |
| 17 | 6,78 | 0,65 | 2,46 | 4,01 | 1,6 | 1 | 0 | 0,96 | 1 |
| 18 | 7,74 | 2,38 | 1,4 | 5,22 | 3,7 | 1 | 0 | 0,93 | 1 |
| 19 | 3,68 | 0,95 | 0,9 | 2,35 | 2,6 | 1 | 0 | 1 | 1 |
| 20 | 6 | 1,5 | 1,9 | 4 | 2,1 | 0 | 0 | 0,7 | 0 |
| 21 | 6,91 | 0,9 | 1,78 | 4,7 | 2,6 | 0 | 0 | 0,58 | 0 |
| 22 | 6,08 | 0,92 | 2,06 | 3,58 | 1,7 | 0 | 0 | 0,83 | 0 |
| 23 | 6,13 | 1,04 | 2,3 | 3,34 | 1,5 | 0 | 0 | 0,67 | 0 |
| 24 | 9,45 | 1,14 | 1,93 | 6,98 | 3,6 | 0 | 0 | 0,75 | 0 |
| 25 | 8,47 | 1,38 | 1,99 | 5,82 | 2,9 | 1 | 0 | 0,73 | 1 |
| 26 | 7,48 | 0,81 | 1,02 | 6,08 | 6 | 1 | 0 | 1,08 | 1 |
| 27 | 7,43 | 1,05 | 2,5 | 4,26 | 1,7 | 2 | 0 | 1,04 | 1 |
| 28 | 5,66 | 0,81 | 2,04 | 3,23 | 1,6 | 0 | 0 | 1,06 | 0 |
| 29 | 7,56 | 0,77 | 1,12 | 6,07 | 5,4 | 1 | 0 | 0,81 | 1 |
| 30 | 7,06 | 2,55 | 1,45 | 4,41 | 3 | 1 | 0 | 0,92 | 1 |
| 31 | 6,52 | 1,31 | 1,48 | 4,42 | 3 | 3 | 1 | 0,94 | 1 |
| 32 | 5,66 | 2,57 | 1,09 | 3,36 | 3,1 | 2 | 0 | 1,06 | 1 |
| 33 | 5,77 | 0,97 | 1,33 | 3,98 | 3 | 1 | 0 | 0,95 | 1 |
| 34 | 6,7 | 1,52 | 1,9 | 4,08 | 2,1 | 2 | 0 | 1,11 | 1 |
| 35 | 7,97 | 1,73 | 1,76 | 5,4 | 3,1 | 3 | 0 | 1,2 | 1 |
| 36 | 7,38 | 3,15 | 1,09 | 4,79 | 4,4 | 2 | 0 | 1,14 | 1 |
| 37 | 5,77 | 1,4 | 1,83 | 3,27 | 1,8 | 2 | 0 | 0,89 | 1 |
| 38 | 5,14 | 0,82 | 0,8 | 3,95 | 4,9 | 0 | 0 | 0,81 | 0 |
| 39 | 5,64 | 0,6 | 0,96 | 4,39 | 4,6 | 1 | 0 | 0,74 | 1 |
| 40 | 4,83 | 0,59 | 2,17 | 2,38 | 1,1 | 0 | 0 | 0,82 | 0 |
| 41 | 6,96 | 2,81 | 1,41 | 4,22 | 3 | 0 | 0 | 0,77 | 0 |
| 42 | 6,65 | 0,79 | 1,51 | 4,75 | 3,1 | 1 | 0 | 0,63 | 1 |
| 43 | 6,39 | 1,34 | 1,68 | 4,08 | 2,4 | 0 | 1 | 0,62 | 0 |
| 44 | 7,4 | 1,47 | 1,34 | 5,35 | 4 | 1 | 0 | 0,68 | 1 |
| 45 | 7,61 | 2,48 | 1,42 | 5,02 | 3,5 | 0 | 0 | 0,66 | 0 |
| 46 | 4,36 | 0,76 | 1,27 | 2,73 | 2,1 | 0 | 0 | 0,54 | 0 |
| 47 | 5,25 | 0,8 | 2,5 | 2,36 | 0,9 | 2 | 1 | 1,05 | 1 |
| 48 | 9,25 | 0,63 | 1,13 | 7,82 | 6,9 | 2 | 0 | 1,14 | 1 |
| 49 | 5,79 | 2,97 | 1,35 | 3,04 | 2,2 | 2 | 0 | 1,29 | 1 |
| 50 | 7,22 | 0,88 | 2,1 | 4,7 | 2,2 | 1 | 0 | 1,04 | 1 |
| 51 | 4,81 | 1,62 | 1,48 | 2,52 | 1,7 | 0 | 0 | 0,68 | 0 |
| 52 | 4,91 | 0,91 | 1,69 | 2,78 | 1,6 | 1 | 0 | 0,76 | 1 |
| 53 | 7,84 | 1,07 | 2,49 | 4,85 | 1,9 | 0 | 0 | 0,59 | 0 |
| 54 | 5,19 | 3,1 | 0,92 | 2,81 | 3,1 | 3 | 0 | 0,95 | 1 |
| 55 | 8,16 | 3,14 | 0,82 | 5,85 | 7,2 | 2 | 0 | 0,97 | 1 |
| 56 | 5,97 | 0,89 | 2,32 | 3,23 | 1,4 | 1 | 0 | 1,29 | 1 |
| 57 | 7,17 | 1,14 | 1,69 | 4,94 | 2,9 | 1 | 0 | 1,21 | 1 |
| 58 | 8,02 | 1,48 | 1,83 | 5,48 | 3 | 1 | 0 | 1,16 | 1 |
| 59 | 9,82 | 1,54 | 2,65 | 6,44 | 2,4 | 1 | 0 | 0,58 | 1 |
| 60 | 5,4 | 1,45 | 1,74 | 2,99 | 1,7 | 1 | 1 | 0,73 | 1 |
